# Supplementary material for: PD-L1/PD-1 Expression in the Treatment of Oral Squamous Cell Carcinoma and Oral Potentially Malignant Disorders: An Overview of Reviews
Source: J Pers Med. 2025 Mar 25;15(4):126. doi: 10.3390/jpm15040126 (PMC12028576; doi:10.3390/jpm15040126)
Supplement: Supplementary file 1 [file jpm-15-00126-s001.zip › jpm-3475360-supplementary.pdf]

Table S 1: Keywords used in our study

Keywords	
**Type of study**	"systematic review*" or meta-analys*
**Study sample**	Human or tissue or "cancer tissue" or "human cell" or "cancer cell"
**Disease/condition**	"Oral cancer" or "Mouth cancer" or "oral malignancy" or "head and neck cancer squamous cell carcinoma" or HNSCC or OSCC or "oral neoplasia*" or "oral tumor*" or "oral tumour*" or "oral squamous cell carcinoma" or "oral neoplasms" or "oral neoplasm" or "Oral potentially malignant disorders" or OPMD or OPMDS or "pre-cancerous lesion" or "oral precancerous lesions" or "oral erythroplakia" or "oral leukoplakia" or "oral lichen planus" or "oral submucous fibrosis" or "proliferative verrucous leukoplakia" or "actinic cheilitis" or "dyskeratosis congenita" or "oral graft versus host disease" or "discoid lupus erythematosus" or "oral lichenoid lesions" or "oral premalignant lesions" or "oral dysplasia"
**Marker**	"PD-L1" OR "PDL1" OR "Programmed death-ligand 1" OR "Programmed death-ligand 1 protein" or "Programmed death-ligand 1" or "CD274" or "cluster of differentiation 274" or "B7 homolog 1" or "B7-H1" or "PD-L1 expression" or "PD-L1 biomarker" or PD-1

Table S2: Risk of bias using AMSTAR 2 for SRMAS studies

Questions	Troiano et al., 2019	He et al., 2020	Lenouvel et al., 2020	Yong-Xin Cui1 and Xian-Shuang Su, 2020	Nocini R et al., 2022
Did the research questions and inclusion criteria for the review include the components of PICO?	Yes	Yes	Yes	Yes	Yes
2. Did the report of the review contain an explicit statement that the review methods were established prior to the conduct of the review and did the report justify any significant deviations from the protocol?	Yes	Partial Yes	Yes	Partial Yes	Partial Yes
3. Did the review authors explain their selection of the study designs for inclusion in the review?	NO	NO	NO	NO	No
4. Did the review authors use a comprehensive literature search strategy?	Partial Yes	Partial Yes	Yes	Partial Yes	Partial Yes
5. Did the review authors perform study selection in duplicate?	Yes	Yes	Yes	YES	yes

6. Did the review authors perform data extraction in duplicate?	Yes	Yes	Yes	YES	yes
7. Did the review authors provide a list of excluded studies and justify the exclusions?	Yes	Yes	Yes	YES	No
8. Did the review authors describe the included studies in adequate detail?	Partial Yes	Partial Yes	Yes	Partial Yes	Partial Yes
9. Did the review authors use a satisfactory technique for assessing the risk of bias (RoB) in individual studies that were included in the review? RCT	Partial Yes	Yes	Yes	YES	No
10. Did the review authors report on the sources of funding for the studies included in the review?	No	No	No	NO	No
11. If meta-analysis was performed did the review authors use appropriate methods for statistical combination of results?	Yes	Yes	Yes	YES	yes
12. If meta-analysis was performed, did	Yes	Yes	No	YES	No

the review authors assess the potential impact of RoB in individual studies on the results of the meta-analysis or other evidence synthesis?					
13. Did the review authors account for RoB in individual studies when interpreting/ discussing the results of the review?	Yes	No	Yes	No	No
14. Did the review authors provide a satisfactory explanation for, and discussion of, any heterogeneity observed in the results of the review?	Yes	No	Yes	No	No
15. If they performed quantitative synthesis did the review authors carry out an adequate investigation of publication bias (small study bias) and discuss its likely impact on the results of the review?	Yes	yes	Yes	YES	No
16. Did the review authors report any	Yes	yes	Yes	YES	yes

potential sources of conflict of interest, including any funding they received for conducting the review?					
